# Chromium supplementation in non-obese non-diabetic subjects is associated with a decline in insulin sensitivity

**DOI:** 10.1186/1472-6823-12-31

**Published:** 2012-11-30

**Authors:** Umesh Masharani, Christine Gjerde, Shelley McCoy, Betty A Maddux, Danielle Hessler, Ira D Goldfine, Jack F Youngren

**Affiliations:** 1 Department of Medicine, Diabetes Center, University of California San Francisco, 400 Parnassus Avenue, San Francisco, CA, 94143-1222, USA; 2 Department of Family Medicine, University of California, San Francisco, CA, USA

## Abstract

**Background:**

The use of chromium supplements is widespread for the prevention and treatment of diabetes mellitus but there are conflicting reports on efficacy, possibly reflecting discrepant effects across different populations. In the present studies, we test the hypothesis that chromium supplementation raises serum chromium levels and correspondingly improves insulin sensitivity.

**Methods:**

A double blind placebo-controlled randomized trial was conducted on 31 non-obese, normoglycemic subjects. After baseline studies, the subjects were randomized to placebo or chromium picolinate 500 μg twice a day. The primary endpoint was change in insulin sensitivity as measured by euglycemic hyperinsulinemic clamp. Pre-specified secondary endpoints included fasting lipids, blood pressure, weight, body composition measured by DXA scan.

**Results:**

After 16 weeks of chromium picolinate therapy there was no significant change in insulin sensitivity between groups (p=0.83). There was, however, a strong association between serum chromium and change in insulin resistance (β = -0.83, p=0.01), where subjects with the highest serum chromium had a worsening of insulin sensitivity. This effect could not be explained by changes in physiological parameters such as body weight, truncal fat and serum lipids with chromium therapy.

**Conclusions:**

Chromium therapy did not improve insulin sensitivity in non-obese normoglycemic individuals. Further, subjects who have high serum chromium levels paradoxically had a decline in insulin sensitivity. Caution therefore should be exercised in recommending the use of this supplement.

**Trial registration:**

The study was registered on the NIH registry (clinicaltrials.gov) and the identifier is NCT00846248

## Background

Chromium is a very commonly used nutritional supplement. In 1996 it was estimated that about 10 million people in the United States took chromium supplements at a cost of $ 150 million dollars per year [1], largely as a result of claims of beneficial effects on insulin action and glucose tolerance [2]. The concept that chromium may have a role in carbohydrate metabolism dates back to the 1950’s with the observation that rats fed a Torula yeast-based diet developed glucose intolerance; and that the intolerance was reversed by concentrates prepared from dried Brewer’s yeast and dried porcine kidney powder. Chromium was identified as the active component in these concentrates [3]. Limited animal data and some *in vitro* data on myoblasts suggested that chromium is a positive regulator of insulin action [4-7]. In the 1970s studies of patients with small bowel syndrome suggested that low chromium levels contributed to glucose intolerance that could be reversed by chromium supplementation [8-10]. However, the actual contribution of altered chromium levels to insulin action and glucose homeostasis in humans is not clear.

A recent study of non-diabetic Saudi men and women reported that insulin resistance in this population was associated with increased urinary excretion of chromium. The investigators hypothesized that higher excretion rates could produce chromium deficiencies that could contribute to insulin resistance [11]. However, with no measures of serum chromium, it was not possible to determine whether increased chromium excretion was a primary defect producing reduced serum chromium, or whether higher excretion resulted from higher serum levels. The authors nonetheless postulated that chromium supplementation might be recommended to prevent or delay the progression of insulin resistance into diabetes.

Most of the current knowledge on the effects of chromium on glucose homeostasis comes from clinical studies examining the effect of chromium supplementation on glucose intolerance and insulin resistance. Results from these studies have been inconclusive, however, with both positive and negative findings – reviewed in [2,12-14]. The presence of multiple confounders in the study design make these discrepant results difficult to interpret, and importantly, most studies lacked any measurement of serum or urine chromium levels. Thus the ability of physiological variance in serum chromium to impact insulin action and glucose homeostasis in humans remains unclear.

The present studies were performed to test the hypothesis that chromium supplementation would raise serum chromium levels and correspondingly improve insulin sensitivity. We therefore performed a double blind placebo controlled clinical trial of chromium picolinate therapy in a non-diabetic, non-obese population, and employed the euglycemic hyperinsulinemic clamp to precisely measure insulin sensitivity. We studied this population because of the presence of a range of insulin sensitivities, the likelihood that insulin resistance could result from multiple factors beyond overt obesity, and the previously established relationship between insulin sensitivity and chromium excretion in non-obese, non-diabetic subjects.

## Methods

### Ethical considerations

All subjects gave informed consent. The protocols and consent forms were approved by the University of California, San Francisco institutional review board and Clinical Research Center where the study was conducted.

### Subjects

Non-obese, non-diabetic, healthy subjects between the ages of 20 and 50 were recruited from the local population. A body mass index (BMI) cutoff of less than 27 was chosen due to the wide range of insulin sensitivity values with no correlation to BMI reported for this population [15]. The inclusion cutoff for Asian Americans was set lower at ≤ 25 because of the increased susceptibility for insulin resistance and type 2 diabetes at lower BMI values in this population [16]. Women were premenopausal. Individuals with diabetes, cardiovascular diseases, HIV and other active infections, thyroid disorders, epilepsy, cancer, hepatitis, cystic fibrosis, sickle cell disease, asthma or renal disease were excluded. Subjects taking glucocorticoids, adrenergic agonists, psychotropic drugs, diuretics, beta blockers, HMG CoA reductase inhibitors, or any other medications known to affect insulin sensitivity, carbohydrate metabolism, or lipid metabolism were excluded.

Exercise and general physical activity pattern were determined using the questionnaire developed by [17]. This questionnaire generates a physical-activity index score from 3 to 15 based on work, sport and leisure time energy expenditure, with each category scored from 1 to 5 (lowest to highest activity level). Subjects with scores greater than 10 (population mean approximately 8.3) were not be enrolled in the study.

Subjects underwent a dietary history at enrollment by the clinical research center nutritionist and were placed on a weight maintenance diet in order to avoid the confounding effect of weight loss on insulin sensitivity. Subjects could not be on nutritional supplements for at least three months prior to enrollment.

### Oral glucose tolerance test

A fasting 75 g oral glucose tolerance test (OGTT) was performed. Subjects with impaired glucose tolerance or impaired fasting glucose were excluded.

### Euglycemic hyperinsulinemic clamp

A euglycemic hyperinsulinemic clamp [18] was performed at baseline and after 16 weeks treatment with chromium picolinate or placebo. A primed-continuous infusion of regular human insulin was administered at a rate of 40 mU/min/m^2^ body surface area for 120 minutes. This insulin infusion rate is sufficient to suppress hepatic glucose production in a normal non-obese non-diabetic population [19,20]. Bedside blood glucose levels were measured at 5 minute intervals and the glucose level was maintained at approximately basal level with a variable infusion of 20% glucose. Glucose disposal values (M/LBM/I) were calculated as mg glucose infused per min per kg lean body mass (LBM) during the steady state period between 90 and 120 minutes divided by steady state insulin (SSI) levels (in μU/ml x 100).

### Blood and urine chemistry

Glucose was determined in whole blood by the glucose oxidase technique (Sigma). Insulin levels were measured by ELISA (Millipore, Billerica, MA).

The urine chromium levels were measured by atomic absorption spectrometry with graphite furnace atomization (AAGF) with Zeeman Background Correction and the serum chromium levels were measured by Inductively Coupled Plasma Mass Spectrometry (ICPMS) with Collision Cell Technology at Quest Diagnostics Nichols Institute (Chantilly VA). For AAGF, the sample was diluted with a “matrix modifier” that helped control the atomization of Chromium at a specific temperature. For ICPMS, the sample was diluted with a weak nitric acid solution. A linear calibration curve was obtained on blank samples and performed before and after the assays. Elevated values were repeated with a new sample set-up to check for contamination issues.

### Measurements of body composition indices

Height was measured with a research center stadiometer. Body weight was recorded. Waist and hip circumferences were measured by a standardized protocol. Body composition was assessed by dual-energy X-ray absorptiometry (DXA).

### Muscle biopsies

Percutaneous muscle biopsies were obtained from the belly of the *vastus lateralis* at time of the euglycemic hyperinsulinemic clamp. Biopsies were obtained both prior to and after 120 minutes of insulin infusion on opposite legs. After local anesthesia, a 5 mm diameter Bergstrom needle was passed through a 7 mm skin incision and subcutaneous tissue, and then advanced approximately 2 cm beyond the muscle fascia. The biopsy (75-100 mg tissue) was obtained with applied suction. The incision was closed with steri-strips and firm pressure applied.

### Muscle RNA preparation

RNA was isolated from frozen muscle tissue using the PureLink™ RNA mini kit with TRisol Reagent (Invitrogen, San Diego, CA). 50 mg of frozen muscle tissue was homogenized in 1 ml TRisol® Reagent using the Precellys 24™ Homogenizer (Omni International, Kennesaw, Georgia). Following the tissue homogenization, 0.2 ml chloroform was added to homogenate, shaken vigorously, incubated at room temperature for 2-3 minutes and microfuged at 12,000 g for 15 sec. 400 μl of the clear top layer was transferred to a microfuge tube, and equal parts of 70% ethanol were added to the tube. 700 μl was loaded onto the PureLink spin cartridge to purify the total RNA as directed in the PureLink RNA manual.

### Human insulin signaling PCR array

The human insulin signaling pathway PCR array (PAHS-030A, SA Biosciences/Qiagen) was used to quantify gene expression in muscle biopsy preparations before and after chromium therapy in 8 subjects. This array profiles the expression of 84 genes coding for insulin receptor-associated proteins (including insulin and receptors, insulin-like growth factors and receptors, SH3/SH2 adapter protein); PI-3 kinase pathway proteins, MAPK pathway proteins; primary target proteins for insulin signaling; and target proteins for PPARγ. Each subject was measured in duplicates both before and after treatment. The PCR reaction was performed using manufacturer instructions. Plots of the two technical replicates against each other as well as hierarchical clustering confirmed that the reproducibility of the PCR array was very high. Five housekeeping genes were used: B2M (beta-2-microglobulin), HPRT1 (hypoxanthine phosphoribosyltransferase 1), RPL13A (ribosomal protein L13a), GAPDH (glyceraldehyde 3- phosphate dehydrogenase) and ACTB (beta-actin). Based on quality control analyses, we chose to normalize PCR cycle counts (C_t_) of the target cDNAs such that all arrays have the same average C_t_ of HPRT1, RPL13A and ACTB. The normalized cycle counts (ΔC_t_) are averaged for each pair of duplicates (ΔC_t_^*^) and gene expressions are calculated as 2^-(ΔCt*)^.

### Chromium or placebo treatment

After completion of baseline tests, subjects were randomized to chromium picolinate or placebo 500 μg twice daily for 16 weeks and the tests repeated. Both the investigators and the subjects were blinded. This daily dose of chromium picolinate was chosen because it was reported in one study that 1000 μg daily dose had greater efficacy than a 200 μg daily dose [21]. The chromium picolinate and placebo were supplied by Nutrition21 Inc. (Purchase, NY 10577). Adherence was assessed by pill count. We also measured fasting chromium levels in serum and spot urine at the end of the study.

### Statistical analysis

Statistical analyses were performed using PASW 18.0 (SPSS Inc., Chicago, IL, USA). After data cleaning and checking distributions to assure that all scores met assumptions, key patient characteristics at baseline were compared across the chromium and control groups using chi-square and t-tests. Between-group differences from pre- to post-assessment on LBM M/I were examined with a repeated measures ANCOVA (RM-ANCOVA) analysis controlling for patient characteristics (age, gender, ethnicity, baseline BMI and triglycerides), followed by pair-wise post-hoc tests. Within the chromium treatment group only, the association between chromium absorption and change in insulin resistance was examined in a multiple regression controlling for the same background patient characteristics. Based on the wide range of urinary and serum chromium levels, and the assumption that high chromium levels reflect greater chromium absorption, patients within the chromium group were divided (median split) into a high and low chromium absorption group. A RM-ANCOVA analysis was repeated to test the group x time interaction on M LBM/ I between patients in the placebo group, low chromium absorbers, and high chromium absorbers. Logistic regression analyses were used to explore potential baseline patient measures as predictors of chromium absorption. Exploratory analyses to test whether pre-post changes in body weight or composition or lipids were associated with chromium levels were performed with ANCOVA.

The group sample size of subjects included in the final data analyses (14 active and 15 placebo) had adequate power (.80) to detect an effect of f=.30 or higher. This is equivalent to a medium/large to large effect on insulin sensitivity and had been previously reported with chromium picolinate therapy [22,23].

Two analyses to test for differential gene expression in the insulin signaling pathway between before and after treatment were performed in “R” [24]:

(i) A two-group unpaired Student’s t-test assuming equal group variances. This is also the test performed by the online ‘RT2 ProfilerT PCR Array Data Analysis’ tool provided by the manufacturer.

(ii) A two-group paired Student’s t-test assuming equal group variances (test not provided by manufacturer).

## Results

The completed sample consisted of 31 participants (16 in the chromium group, 15 in the control group). As seen in Table 1, the mean age of the sample was 37 ± 11 years and 55% were male. The majority of participants identified their race as European American/White (58%), 19% as African American, 16% as Asian, and 7% as Hispanic. The average BMI was 23 ± 3 and average fasting glucose was 4.8 ± 0.3 mmol/l.

**Table 1:** Sample description by patient group

	**Total**	**Placebo**	**Chromium**	**t-test or χ**^**2**^
	**N=31**	**N=15 (48.4%)**	**N=16 (51.6%)**	**p-value**
*Patient Characteristics*				
Age (yrs)	37.23 (10.97)	38.60 (10.58)	35.94 (11.53)	.51
Gender				.87
Male	17 (54.8%)	8 (53.5%)	9 (56.3%)	--
Female	14 (45.2%)	7 (46.7%)	7 (43.8%)	--
Ethnicity				.15
Non-Hispanic white	18 (58.1%)	7 (46.7%)	11 (68.8%)	--
African American	6 (19.4%)	4 (26.7%)	2 (12.5%)	--
Asian	5 (16.1%)	4 (26.7%)	1 (6.3%)	--
Hispanic	2 (6.5%)	0 (0%)	2 (12.5%)	--
BMI	23.12 (3.09)	22.68 (2.26)	22.53 (3.74)	.45
Waist circumference (cm)	79.54 (13.05)	78.7 (6.93)	80.27 (17.15)	.75
Fasting Glucose (mmol/L)	4.81 (0.29)	4.40 (0.27)	4.55 (0.29)	.14
Total cholesterol (mmol/L)	4.49 (0.13)	4.41 (0.86)	4.56 (0.82)	.61
Triglycerides (mmol/L)	0.91 (0.40)	0.82 (0.17)	1.01 (0.53)	.21
LDL cholesterol (mmol/L)	2.25 (0.55)	2.15 (0.45)	2.34 (0.63)	.35
HDL cholesterol (mmol/L)	1.51 (0.40)	1.6 (0.44)	1.43 (0.35)	.24

Patients in the intervention group did not statistically differ from the control group on any patient characteristics at baseline (Table 1). The subjects tolerated the chromium or placebo and there were no adverse reactions reported. Because of previous reports that chromium therapy may impair renal function [25,26], subjects underwent interval measurements of electrolytes and creatinine which were unchanged. After determining that two subjects in the chromium group did not comply with taking their chromium as directed (based on serum and urine chromium levels) they were eliminated from all further analyses, for a total sample of 29 patients (14 in the chromium group and 15 in the control group). Keeping the two subjects who did not comply with chromium therapy in the analyses did not alter the conclusions. There was no change in BMI or truncal fat or lipid levels in response to treatment. Diet and physical activity as measured by questionnaire stayed constant throughout the study.

### Relationship between chromium and insulin sensitivity at baseline

At baseline, subjects in the placebo group and chromium group had very low urine chromium levels (range 0-0.5 μ/g creatinine) and serum chromium levels (range <0.5 to 0.6 μg/L). The relation between insulin sensitivity and both urinary and serum chromium levels was not statistically significant (r = 0.24, p=0.1; r=0.08, p=0.79 respectively).

### Changes in insulin sensitivity by chromium absorption

**Figure 1:**
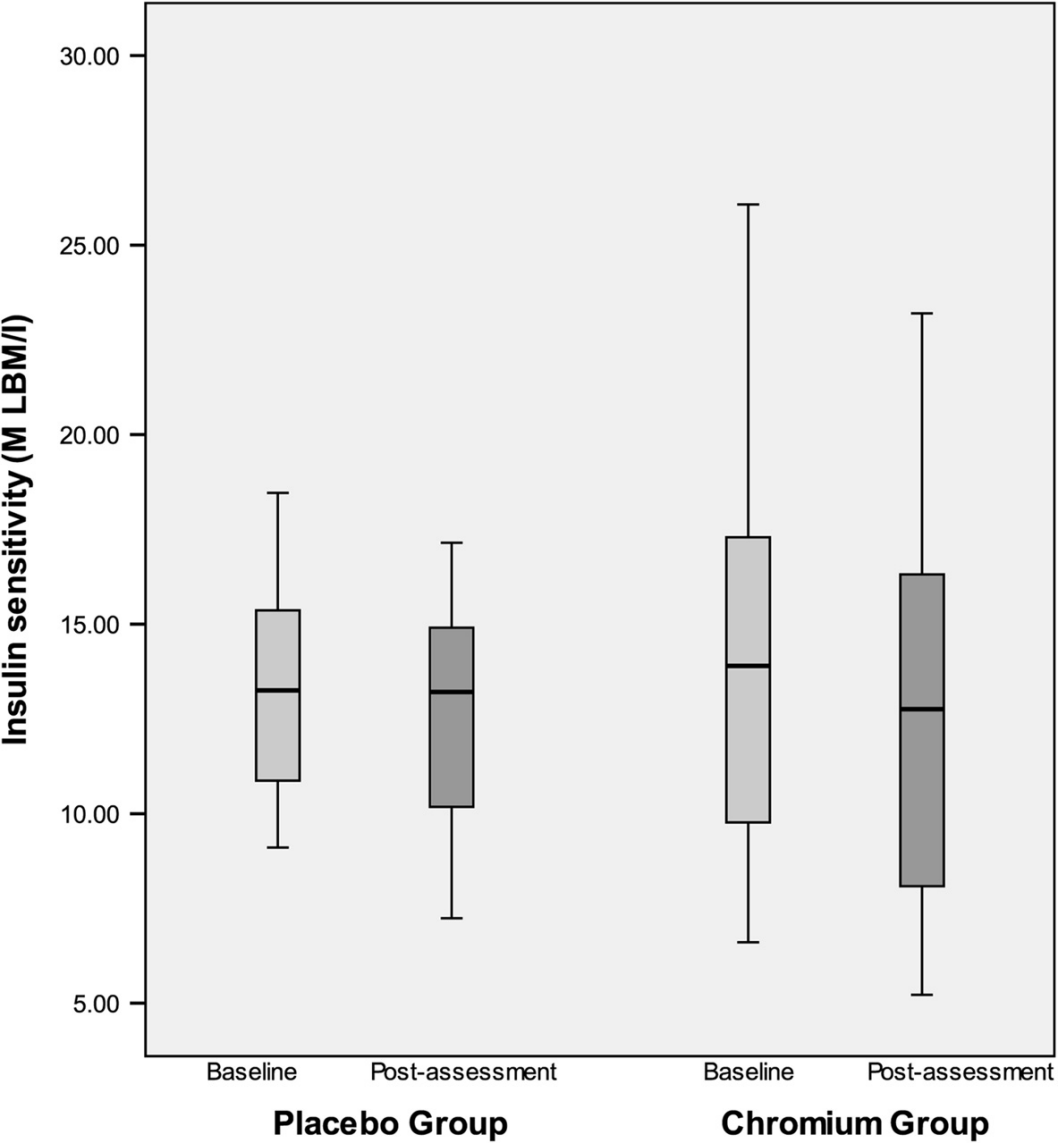
**Boxplot of pre and post insulin sensitivity as measured by euglycemic hyperinsulinemic clamp (M LBM/I) by group (0 = placebo group, 1 =chromium group) after 16 weeks of chromium picolinate versus placebo.** There was no significant change in insulin sensitivity. -1.63 mg/min/kg/mU insulin (-8.9 to + 5.57) in the placebo group and -1.14 mg/min/kg/mU insulin (-5.22 to + 4.11) in the chromium group (p=0.83). The serum chromium levels ranged from less than 0.5 to 1.0 μg/L in the placebo group and 0.8 to 5.5 μg/L (mean 3.0) in the chromium treated group.

Insulin sensitivity (M LBM/I) did not significantly differ in the chromium and placebo groups at baseline (t= -0.22, p=0.83). After 16 weeks of therapy, the mean change in insulin sensitivity was -1.63 mg/min/kg/mU insulin (range: -8.9 to + 5.57) in the placebo group and -1.14 (-5.22 to + 4.11) in the chromium group (see Figure 1). The group x time interaction was not significant [F(1,22) = 0.36, p=.54] suggesting the degree of change in insulin sensitivity from pre- to post-assessment did not statistically differ by group.

**Figure 2:**
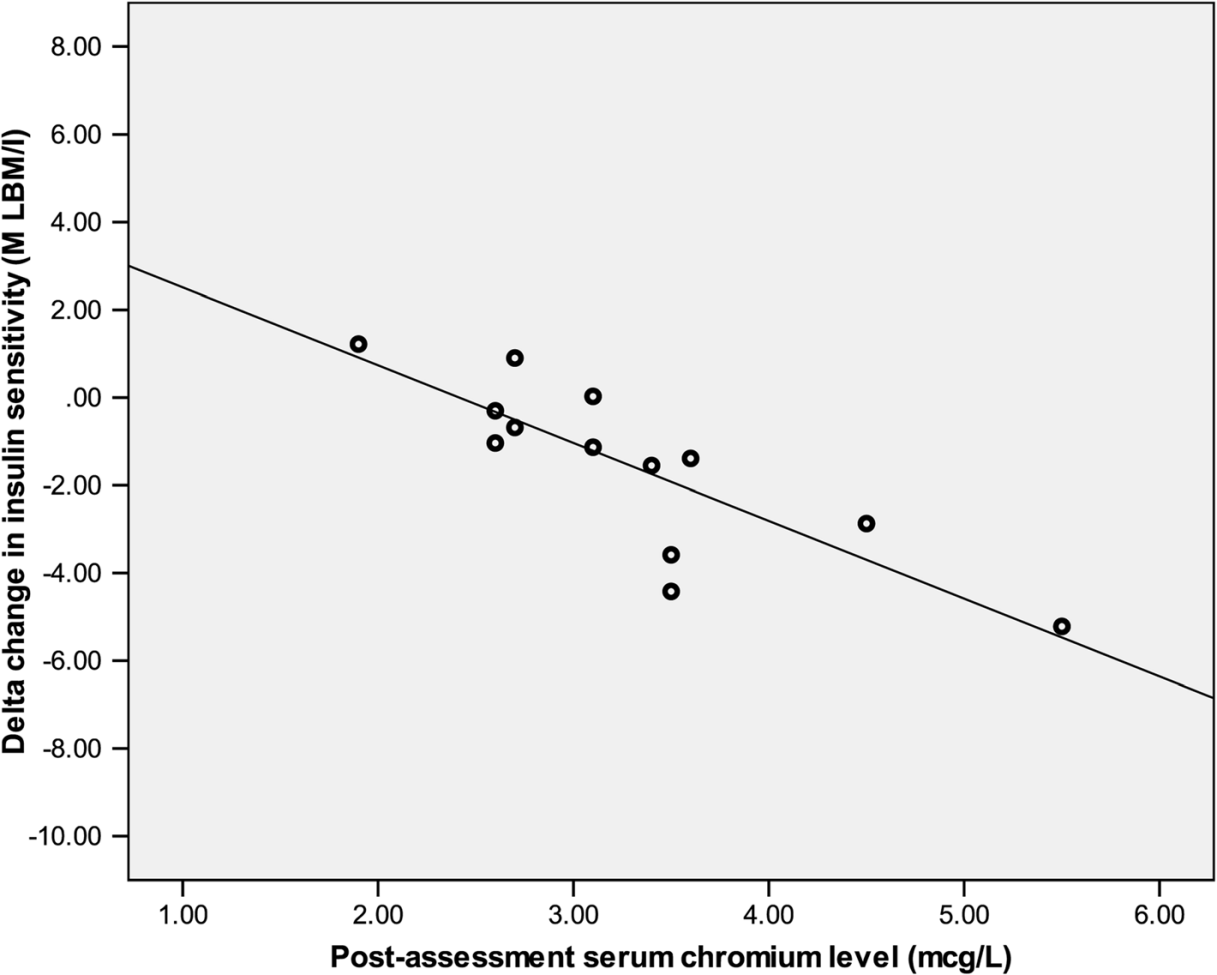
**Scatterplot of the delta change in insulin sensitivity (M LBM/I) chromium group against fasting serum chromium levels at 16 weeks.** High serum chromium levels are associated with increase in insulin resistance, r=0.85, p=0.01.

**Figure 3:**
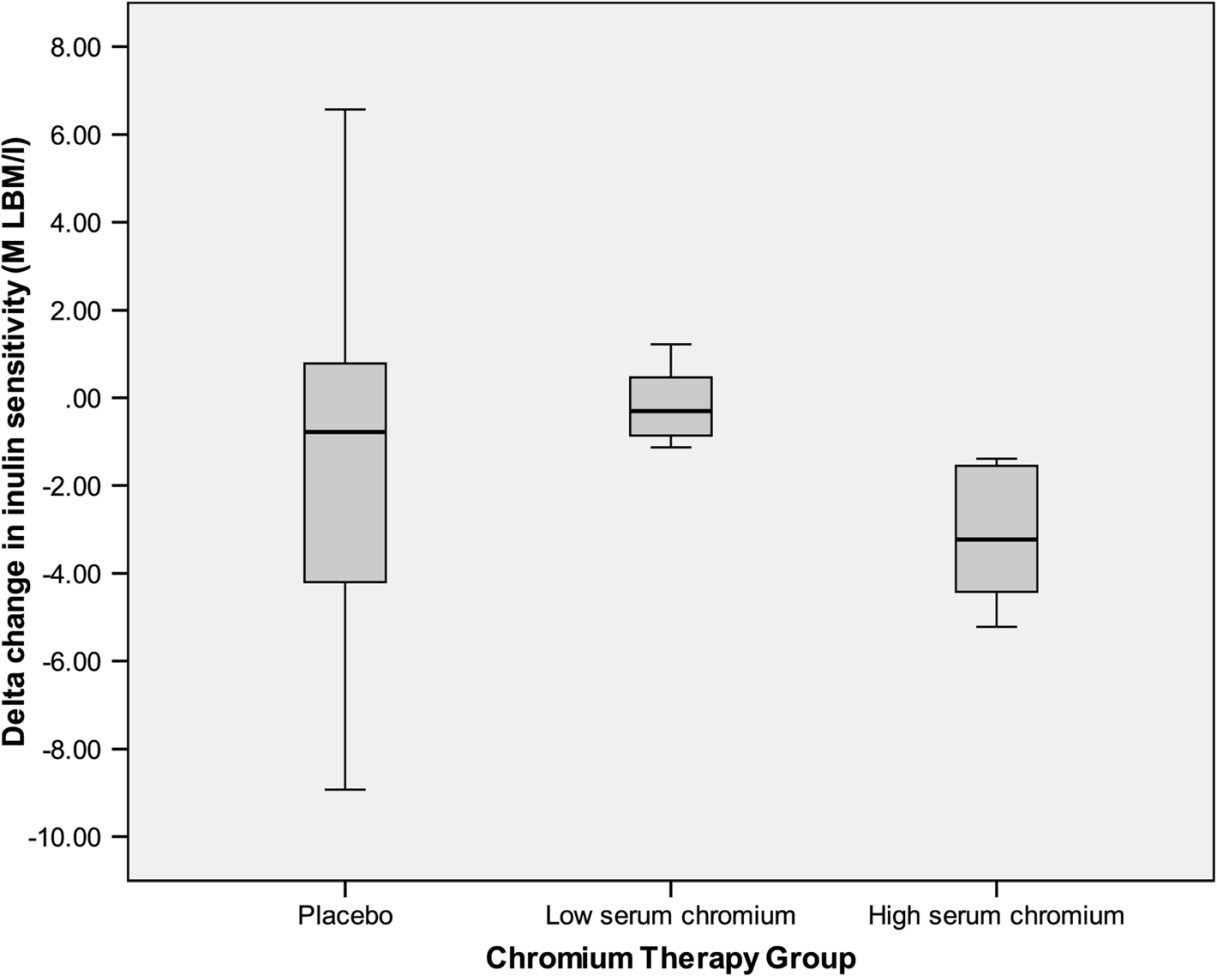
**Boxplot of the delta change in insulin sensitivity (M LBM/I by group: Chromium therapy group is divided into high serum chromium group (>3.1 μg/L); low serum chromium group (≤ 3.1 μg/L); and placebo group.** High serum chromium group had had greater insulin resistance than participants in the low serum chromium group or the placebo group, p=0.02, p=0.05 respectively.

Among the chromium treated subjects there was a wide range of serum and urinary chromium values following treatment. Urine chromium levels ranged from 2.8 to 15.9 μg/g creatinine and the serum chromium levels ranged from 0.8 to 5.5 μg/L for participants within the chromium group. Post treatment serum chromium levels were highly correlated with urine chromium excretion (r=0.89, p<0.01). Neither serum nor urinary chromium levels changed in the placebo group (data not shown). Due to the apparent variation in the degree of chromium absorption between subjects, we examined the relationship between serum chromium and change in insulin resistance. After controlling for baseline patient characteristics, results of a multiple regression analysis showed a strong association between serum chromium and worsening of insulin–mediated glucose disposal (β = -0.83, p<0.01), where subjects with the highest serum chromium had a *decline in their insulin sensitivity* (Figure 2). To further explore the association between chromium absorption and insulin resistance, patients within the chromium group were divided (based on a medial split at 3.10 μg/L) into a high (n=6) and low (n=8) serum chromium group. In a RM-ANCOVA analysis there was a significant time x group interaction [F(2, 21) = 3.55, p=0.05]. There were no group differences at baseline; however, at post-assessment participants in the high serum chromium group (> 3.1 μg/L) were more insulin resistant than participants in the low serum chromium group (≤ 3.1 μg/L) or the placebo group (p=0.02, p=0.05 respectively) (Figure 3). Insulin sensitivity did not differ between the placebo and low serum chromium groups (p=0.25).

To explore whether the association between chromium absorption level and change in insulin sensitivity is explained by treatment-mediated changes in other key variables, we examined associations between chromium absorption levels with pre- post changes in triglycerides (F=.48, p=.51), LDL, (F=.07, p=.80) BMI, (F=3.14, p=.13), and truncal fat (F=1.11, p=.34) and results were non-significant. Furthermore, when changes in triglycerides, LDL, BMI, and truncal fat were individually added to the model, none were independent significant predictors of change in insulin sensitivity, and chromium absorption remained a significant predictor of reduced insulin sensitivity in each model. These results suggest, therefore, a direct effect of chromium on changes in insulin action that are independent of lipids and adiposity.

### Predictors of chromium absorption

In exploratory analyses, we examined potential baseline predictors of high vs. low chromium absorption (among only patients in the chromium group) with logistic regression analysis. While no baseline variables reached statistical significance, the largest effects were for participants who were older (OR=1.09, p=0.17), those with lower triglycerides (OR=0.95, p=0.18), and those with lower levels of homocysteine (OR=0.38, p=0.13) to have a greater likelihood of being in the high absorption group.

### Human insulin signaling PCR array analysis

In order to explore the possibility that high serum chromium levels could have a direct effect on skeletal muscle, the primary site for insulin-mediated glucose disposal, we analyzed skeletal muscle biopsies from 4 subjects who had high serum chromium levels and became insulin resistant and 4 subjects who had lower serum chromium levels and had no change in insulin sensitivity. Differential expression of genes in the insulin signaling pathway was explored for high- and low-chromium subjects by PCR array as evidence for a direct impact of chromium on skeletal muscle.

At a 1% significance level, both unpaired and paired analyses failed to identify any significant change in expression of genes in the insulin signaling pathway with chromium therapy.

## Discussion

We studied the effect of chromium picolinate on insulin-stimulated glucose uptake in a well-characterized population of non-obese non-diabetic subjects. We did not find evidence that serum chromium levels had a significant impact on insulin sensitivity in this population, nor did we find evidence that high chromium excretion was associated with insulin resistance. In investigating the use of daily chromium picolinate supplementation as a modulator of insulin action, we could find no evidence for a beneficial effect of this compound. Our principal finding was that those subjects with the highest levels of serum chromium following treatment had worsening of insulin sensitivity rather than an improvement. This negative effect of chromium supplementation was highlighted when we divided the chromium treated subjects into two groups using the medial serum chromium value of 3.1 μg/L – the subjects with the higher chromium levels had greater reduction in insulin sensitivity than the lower chromium levels. There were no obvious baseline characteristics that identified individuals who were likely to have greater serum levels of chromium. The increase in insulin resistance could not explained by weight gain or alterations in lipid levels.

While claims of benefits of chromium therapy on weight loss and increased muscle mass have been largely discredited [2,27], this supplement is still being promoted for its beneficial effects on insulin action and glucose tolerance [2]. In the 1970’s and 1980’s there were three clinical reports of [8-10] of patients with small bowel loop syndromes on parental nutrition who developed glucose intolerance and who improved following chromium therapy. These case reports led to the routine addition of chromium to TPN solutions. They also provided impetus for other clinical studies (see reviews [2,12,13]) of chromium therapy in normal subjects, people with glucose intolerance, and people with diabetes (both type 1 and type 2) [21-23,28-46]. The results have been inconclusive, with some studies reporting a positive effect on glucose and/or insulin levels whereas others failing to show benefit. Design problems include open label studies [22,30,35,39]; some studies including subjects with different disorders s e.g. type 1 and type 2 patients [30,34]; type 2 subjects remaining on drugs that affect insulin secretion or insulin action [35,39,47]; inadequate control of confounders such as hyperglycemia and obesity that affect insulin action [22]; and failure to address the role of chromium levels on the measured parameters. A significant limitation of most of these studies is their use of surrogate measures such as fasting insulin and glucose tolerance tests which may not be sensitive enough to assess changes in insulin action with therapy. Very few studies employed the more accurate euglycemic hyperinsulinemic clamp technique to assess insulin action [23,42]. The studies also used a wide range of chromium doses, formulations and durations of therapy. Supplementation with 200 to 1000 μg of chromium picolinate has been reported to improve glucose intolerance and lower circulating insulin levels [21,48] with one study in type 2 diabetes patients showing a greater improvement with 1000 μg dose compared to 200 μg dose [21]. The 1000 μg daily has been used in a number of other clinical studies [28,29] and not associated with any toxic effects.

We have addressed many of these limitations in the present study. The subjects had normal glucose intolerance and were of normal weight. The absence of obesity and hyperglycemia removes two important confounders that can affect insulin action. We also measured serum and urine chromium levels to confirm compliance and also to assess effects of chromium levels on the measured parameters. We used the euglycemic hyperinsulinemic clamp rather than surrogate measures to assess insulin action before and after therapy. We also used chromium picolinate at a dose that previously had been reported to improve insulin resistance.

We did not observe an increase in insulin sensitivity in the treatment group, but observed a decrease in insulin sensitivity only in subjects who had high levels of serum or spot urinary chromium levels. Generally, the absorption of chromium picolinate has reported to be very low in the range of 2.8 ±1.14% (SD) but there is significant individual variation with peak urinary excretion varying 5 fold after acute dosing [49]. Distribution studies with labeled chromium also suggest that trivalent chromium is stored in the body principally in the liver and also in kidneys, spleen and muscle [2] and it is possible that the impact of chromium therapy may only become apparent in studies lasting several months.

In order to further explore the mechanism by which chromium picolinate induces insulin resistance, we used a human insulin signaling pathway PCR array to examine the expression of genes in skeletal muscle before and after chromium therapy. We were unable to demonstrate any changes in gene expression with therapy in 4 subjects who became insulin resistant with therapy. The expression array examines only the genes coding for proteins that have been directly implicated in, or affected by, insulin signaling. It is of course possible that chromium therapy affects the expression of other genes that were not studied; or that chromium affects muscle insulin signaling at the level of protein function, without an impact on expression of insulin-sensitive genes, through actions that may or may not originate in skeletal muscle. Proposed targets for chromium action include membrane phosphotyrosine phosphatase [50], insulin receptor tyrosine kinase [51], protein-tyrosine phosphatase 1B [52].

Some additional limitations of our study need to be discussed. The original study was designed to evaluate group differences (placebo vs. chromium therapy) and not powered to examine subgroups (serum chromium levels). The results therefore should be viewed with some caution and need to be replicated. We restricted our recruitment to normoglycemic and non-obese subjects and it remains possible that people with glucose intolerance or frank diabetes may respond differently, due to effects of chromium on insulin secretion or glucose toxicity. Our subjects were healthy eating a normal diet and so were not chromium deficient. It is quite possible as noted in the case reports of short bowel syndrome patients on TPN in the 1970’s that extreme chromium deficiency causes defects in insulin secretion and/or action. The concept that extreme deficiency or excess of an agent can have the same clinical effect is not without precedence. Both magnesium deficiency and excess inhibits parathyroid hormone secretion resulting in hypocalcemia; and the hypocalcemia due to deficiency is corrected by magnesium supplements.

## Conclusions

In conclusion, pharmacological doses of chromium picolinate therapy do not improve insulin sensitivity in normal non-diabetic subjects. Indeed, it appears that subjects for whom chromium supplementation produced high serum chromium levels paradoxically had a worsening of their insulin sensitivity. We do not have information regarding the mechanism by which high serum chromium levels decreases insulin sensitivity. We can say that it is unlikely to be due changes in expressions of genes that have been identified to be involved in the insulin signaling pathway.

If this finding of worsening insulin sensitivity with high serum chromium levels is confirmed then it is possible that the widespread use of chromium supplements may be contributing to, rather than ameliorating, insulin resistance in the population.

## Competing interests

The authors do not have any relevant conflicts of interest.

## Authors’ contributions

UM, IDG, JFY conceived and designed the experiments. UM, CG, SM, BAM performed the experiments. UM, DH, JFY analyzed the data. UM, DH, IDG, JFY wrote the manuscript. All authors read and approved the final manuscript.

## Pre-publication history

The pre-publication history for this paper can be accessed here:

http://www.biomedcentral.com/1472-6823/12/31/prepub
